# HSPA1A, HSPA2, and HSPA8 Are Potential Molecular Biomarkers for Prognosis among HSP70 Family in Alzheimer's Disease

**DOI:** 10.1155/2022/9480398

**Published:** 2022-09-30

**Authors:** Yeqing Dong, Tongxin Li, Zhonghui Ma, Chi Zhou, Xinxu Wang, Jie Li

**Affiliations:** ^1^Institute of Mental Health, Tianjin Anding Hospital, Mental Health Center of Tianjin Medical University, Tianjin 300222, China; ^2^Department of Laboratory Medicine, Tianjin Anding Hospital, Mental Health Center of Tianjin Medical University, Tianjin 300222, China; ^3^Laboratory of Biological Psychiatry, Institute of Mental Health, Tianjin Anding Hospital, Mental Health Center of Tianjin Medical University, Tianjin 300222, China

## Abstract

Alzheimer's disease (AD) is a chronic neurodegenerative disease, which leads to impairment of cognition and memory. The heat shock protein 70 (HSP70) family plays an important role in the pathogenesis of AD. It is known to regulate protein misfolding in a variety of diseases, including inhibition of A*β* aggregation and NFT formation in AD. As yet, the diagnostic molecular markers of AD remain unclear. Herein, we sought to investigate molecular markers of HSP70 family that can affect diagnosis and treatment in AD through computational analysis. In this study, the intersection between HSP70 family members and immune molecules was taken to screen immune-related HSP70 family genes. Based on the datasets from the NCBI-Gene Expression Omnibus (GEO) database, we found that the expression levels of HSPA1A and HSPA2 were significantly increased in AD samples, while HSPA8 significantly decreased. Surprisingly, the combination of the 3 hub genes had a good diagnosis of AD via receiver operating characteristic curve (ROC). Moreover, the clinical value of the 3 hub genes was further assessed by the Spearman correlation analysis with AD-related genes, *β*-secretase activity, and *γ*-secretase activity. In terms of immune cell infiltration, we showed that the distribution of seven immune cell types (macrophages M2, neutrophils, T cells CD4 memory activated, macrophages M0, NK cells activated, plasma cells, and T cells follicular helper) was associated with the occurrence of AD by CIBERSORT. Furthermore, our data suggested that EP300, MYC, TP53, JUN, CREBBP, and ESR1 might be key transcription factors (TFs) for the 3 hub genes. In general, these findings suggest that HSPA1A, HSPA2, and HSPA8 are potential molecular biomarkers for prognosis among HSP70 family in AD, and it provides a new perspective on diagnostic and therapeutic targets for AD.

## 1. Introduction

It is estimated that 47 million people live with dementia globally, and it will increase more than threefold (~131 million) by 2050 [1]. Alzheimer's disease (AD) is a common neurodegenerative disease, which is the major cause of dementia [2]. It will reduce the life quality of patients, affect the normal life of their families, and increase the cost burdens of care [3]. Currently, it has evolved into one of the great health-care challenges of the twenty-first century [4]. The pathogenesis of AD has been attributed to extracellular aggregates of amyloid *β* (A*β*) plaques and intracellular neurofibrillary tangles formed by hyperphosphorylated *τ*-protein (tau) in the human brain [1]. It is characterized by memory impairment and progressive neurocognitive dysfunction [5]. Some studies have shown that it also involves disturbances of mitochondria, abnormalities of lipid metabolism, oxidative stress, immune system and inflammatory responses, and other pathways [6, 7]. For instance, oxidative stress and neuroinflammation ultimately increase neuronal death through apoptosis or other mechanisms, and they are responsible for the behavioral deficits including AD [8]. They produce some of the insidious effects, including cognitive dysfunctions such as learning and memory impairment [9, 10]. In terms of AD-related molecular markers, APOE gene has been shown to be closely associated with the incidence of AD in most cases [11]. Other related genes include mutations in amyloid protein precursor (APP), presenilin-1 (PSEN1) and presenilin-2 (PSEN2) that make individuals susceptible to AD [12]. However, the pathogenesis and diagnostic molecular markers of AD and remains largely unclear. In particular, little is known about the immune aspects of AD. Therefore, it is of great significance to study the underlying molecular mechanisms and identify more reliable molecular markers of AD.

It is well known that misfolding protein aggregation in the human brain is one of the key features of many neurodegenerative diseases [13]. And the main hallmark in AD is the formation of A*β* plaques and tau protein aggregates [14]. The heat shock protein (HSP) family, particularly HSP70, plays an important role in this process and is known to regulate protein misfolding, including tau levels and toxicity in AD. The HSP70 family, consisting of 17 members, is a class of highly abundant and widely expressed chaperone proteins [13, 15]. They are encoded by a multigene family encompassing up to 17 genes and 30 pseudogenes [16]. In terms of function, the HSP70 family members are involved in numerous biological processes, including nascent polypeptide folding, protein trafficking, the refolding or degradation of aggregated peptides, and an immunomodulatory effect [17, 18]. In AD, it has been shown that HSP70 colocalizes with A*β* plaques and takes part in the neuroprotective response to suppress A*β* aggregation [19–21]. HSP70 also promotes solubility and the ability to bind microtubules of tau, hence inhibiting formation of neurofibrillary tangles [22]. In addition, an exon microarray data showed that HSPA1A was found to be upregulated in patients with AD, and it might be involved in protein folding abnormality and altered synaptic transmission on the pathomechanism of AD [23]. HSPA2 elevated A*β*40 and A*β*42 levels and phosphorylated-tau in two cell lines with HSPA2 overexpression and was nominated HSPA2 as a specific key regulator of late-onset Alzheimer's disease [24]. These evidences manifest that the HSP70 family plays a major role in AD's pathogenesis. However, at present, studies on the role of HSP70 family members in the pathogenesis of AD are scattered, only targeting a specific gene, such as HSPA1A. Considering the involvement of processes in AD etiology, targeting all members of the HSP70 family is a promising diagnostic and therapeutic prospect.

**Figure 1:**
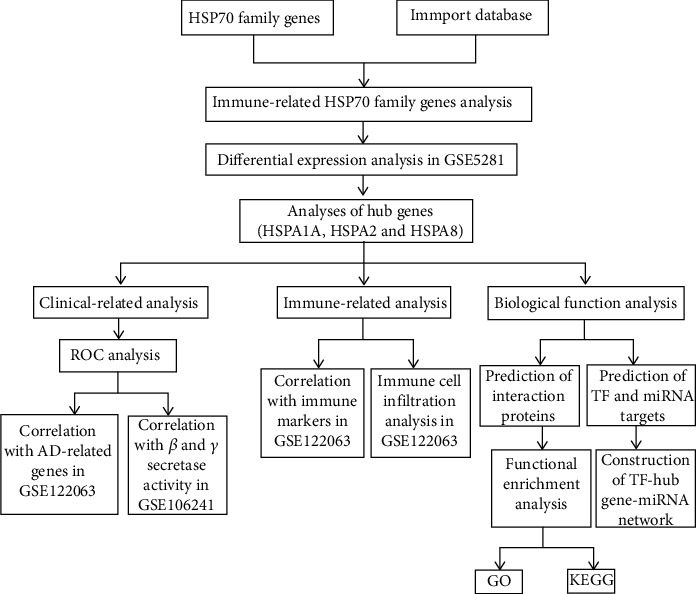
Workflow of computational analysis of immune-related HSP70 family members in AD.

In this study, after integrating combined analysis between HSP70 family members and immune molecules in the ImmPort database and differential expression analysis, we preliminarily identified HSPA1A, HSPA2, and HSPA8 as hub genes of immune-related HSP70 family in AD. An in-depth analysis of the 3 hub genes functions in AD patients based on the datasets from the GEO database published online to determine their distinct diagnostic values and the potential functions in AD (Figure 1). To our knowledge, this is the first report that screens HSP70 family members to identify a series of hub genes in AD and performs clinical-related analysis, immune-related analysis, and biological function analysis of hub genes. Our data reveals a novel understanding of AD pathogenesis in HSP70 family and provides a set of potential useful targets for further research on molecular mechanisms and diagnostic biomarkers.

## 2. Materials and Methods

### 2.1. Data Collection

Immune molecules data were retrieved from the ImmPort database [25] (https://www.immport.org/) (Supplementary Table 1). FunRich software [26] is user-friendly and provides graphical representation of the data. It was used to take intersection for screening candidate immune-related HSP70 family genes. The Gene Expression Omnibus [27] (GEO, http://www.ncbi.nlm.nih.gov/geo) is a public, free, and easy-to-use database with a large amount of data for researchers. We searched the GEO database for microarray datasets using the keyword “Alzheimer's disease.” Datasets were included if they met the following criteria: (1) were from humans; (2) included expression data from the brain regions of both AD and control samples and expression data from the brain regions of AD samples with *β*/*γ* secretase activity; (3) the number of AD samples was ≥50, and the number of control samples was ≥40; and (4) were downloaded to analysis. Finally, three datasets were obtained (GSE5281 [28], GSE132903 [29], and GSE122063 [30], including AD and control samples); one dataset (GSE106241 [31]) from AD samples with *β*/*γ* secretase activity was selected. Because GSE5281 (AD: 84, control: 74) and GSE132903 (AD: 97, control: 98) had far more samples, the array data of GSE5281 were used for differential expression analysis between the AD and control groups, and GSE132903 was used to validate further the differential expression of hub genes. The array data of GSE122063 was used for correlation with AD-related genes and immune-related analysis. The array data of GSE106241 was used for the correlation with *β* and *γ* secretase activity.

### 2.2. Disease-Diagnosing Ability and Correlation Analysis

To assess the diagnostic role of hub genes, we performed the receiver operating characteristic curve (ROC) analysis by SPSS 18.0 [32] (SPSS Inc., Chicago, USA). Moreover, we evaluated the Spearman correlation analysis between the 3 hub genes and AD-related genes, *β*-secretase activity and *γ*-secretase activity to understand whether the hub genes could reflect the clinical significance in AD.

### 2.3. Immune Cell Infiltration Analysis

To understand the status of the immune microenvironment in AD patients, we carried out CIBERSORT R package [33] of 110 samples in GSE122063, which was a robust algorithm for calculating the cellular composition of a tissue. The LM22 (22 immune cell types) was downloaded to act as a reference gene expression signature. Finally, Pearson correlation coefficient was utilized to evaluate the correlation with immune cell infiltration.

### 2.4. Prediction of Interaction Proteins and Functional Enrichment Analysis

GeneMANIA [34] (http://www.genemania.org) is a flexible, fast updated, and user-friendly website, which can provide interactions of proteins and genes and a variety of functions of submitted genes. To predict the interaction proteins of the 3 hub genes, we utilized GeneMANIA and exported the visualization results. Metascape [35] (http://metascape.org) is a practical, visual tool to perform gene annotation and pathway enrichment analysis. In terms of biological functions, the 3 hub genes, predicted interaction genes, and star molecules in AD were analyzed using Metascape, including the gene ontology (GO) enrichment and KOBAS-Kyoto Encyclopedia of Genes and Genomes (KEGG) pathways analysis.

### 2.5. Construction of TF-Hub Gene-miRNA Network

The hTFtarget [36] (http://bioinfo.life.hust.edu.cn/hTFtarget#!/) was utilized to predict transcription factors (TFs) of hub genes. Then, the intersection of predicted TFs was taken via Jvenn website [37] (http://jvenn.toulouse.inra.fr/app/example.html), and the CytoHubba plug-in was used to select the TFs with high scores in Cytoscape [38]. The miRNAs of hub genes were predicted by using two online databases, TargetScan [39] (http://www.targetscan.org/) and miRDB [40] (http://mirdb.org/), which are commonly used to predict miRNAs. The miRNAs predicted by these two programs overlapped were identified as miRNA targets, in which the miRNA score predicted by the miRDB was over 80. Finally, the TF-hub gene-miRNA network was constructed using the Cytoscape software.

### 2.6. Statistical Analysis

Data obtained from GEO database were analyzed through Student's *t*-test. GraphPad Prism version 6.00 [41] was used for differential expression and correlation analysis, and values were expressed as Mean ± SD. *P* < 0.05 was considered statistically significant.

## 3. Results

### 3.1. Screening and Identification of Hub Genes by Computational Analysis

**Figure 2:**
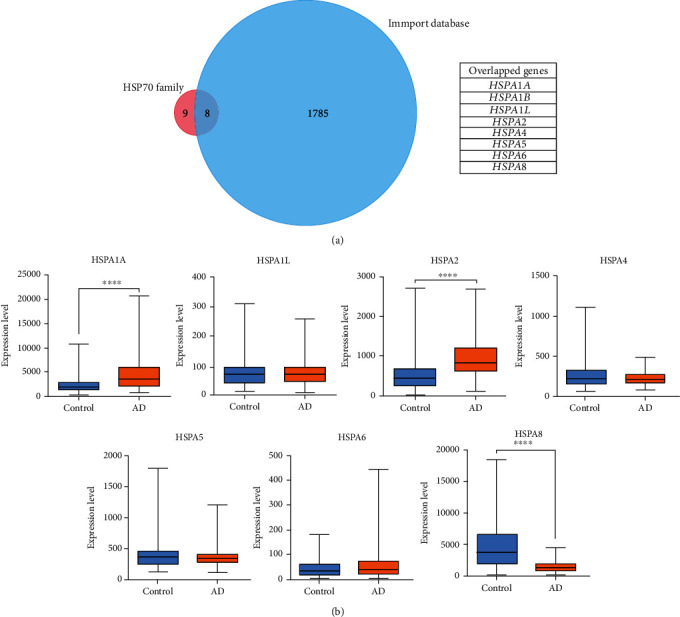
Screening and identification of hub genes by computational analysis. (a) Immune-related HSP70 family genes between the HSP70 family members and immune molecules in the ImmPort database were screened by Venn diagrams. The HSP70 family and the ImmPort database contain 17 and 1793 molecules, respectively, of which 8 are common between both groups. (b) The expression levels of seven immune-related HSP70 family members in GSE5281, except HSPA1B. The blue box indicates the control group, and the orange box indicates the AD group. Data were analyzed by Student's *t*-test and expressed as the Mean ± SD (AD: 84, control: 74). ∗∗∗∗ (*P* < 0.0001) represents significance compared to the control group.

The 17 members of the HSP70 family (Table 1) and immune molecules in the ImmPort database were screened to take intersection for identifying immune-related HSP70 family genes using FunRich software. A total of 8 overlapped genes were obtained (Figure 2(a)). Furthermore, 87 AD samples and 74 control samples of GSE5281 were used for differential expression analysis of immune-related HSP70 family genes by GraphPad Prism 6.00 software. The results showed that HSPA1A and HSPA2 were significantly increased in AD group when compared with the control group (*P* < 0.0001 and *P* < 0.0001, respectively, Figure 2(b)), while HSPA8 decreased significantly (*P* < 0.0001, Figure 2(b)). It is worth mentioning that the HSPA1B gene was not clearly matched in the dataset, so it was excluded in the subsequent analysis. We have also analyzed GSE132903 dataset (AD: 97, Control: 98), and the verification results showed that the expression level trend of HSP1A, HSPA2, and HSPA8 gene was consistent (*P* < 0.05, *P* < 0.05, and *P* < 0.001, respectively, Supplementary Figure 1). Finally, we preliminary identified HSPA1A, HSPA2, and HSPA8 as hub genes of immune-related HSP70 family.

### 3.2. ROC and Correlation Analyses Reveal Potential Clinical Values of Hub Genes

**Figure 3:**
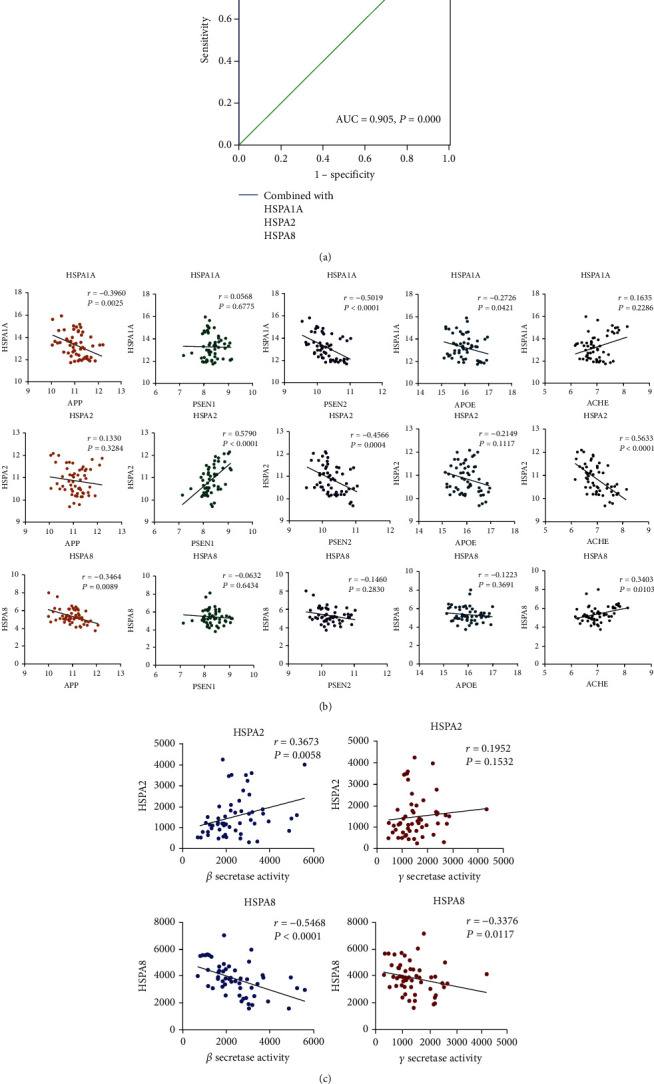
ROC and correlation analyses reveal potential clinical values of hub genes. (a) ROC curve in GSE122063 for distinction of AD and control based on the combination of HSPA1A, HSPA2, and HSPA8 (AD: 56, control: 44). (b) Correlation analysis between the 3 hub genes (HSPA1A, HSPA2, and HSPA8) and AD-related genes (APP, PSEN1, PSEN2, APOE, and ACHE) in GSE122063 (*n* = 56). Data were analyzed by the Spearman correlation analysis. (c) Correlation analysis to determine the clinical values between the hub genes (HSPA2 and HSPA8, except HSPA1A) and *β*-secretase activity and *γ*-secretase activity in GSE106241 (*n* = 60). Data were analyzed by the Spearman correlation analysis. For panels (b) and (c), *P* values in red are significant (*P* < 0.05).

ROC analysis was performed to assess the diagnostic role of hub genes by SPSS 18.0. Surprisingly, we found that the combination of HSPA1A, HSPA2, and HSPA8 had a good ability to differentiate the AD (*n* = 56) and control (*n* = 44) groups in GSE122063 (Figure 3(a)). The area under the curve (AUC) for differentiating AD and control samples is 0.905 [95%confidence interval (CI) = 0.847–0.963]. Next, GSE122063 was also chosen for correlation analysis between hub genes and AD-related genes. Previous studies have shown that familial autosomal dominant AD genes amyloid protein precursor (APP) presenilin-1 (PSEN1), presenilin-2 (PSEN2), and major genetic risk factor APOE have been elucidated as the AD-related genes [42]. We found that HSPA1A and HSPA8 were negatively associated with APP (*P* = 0.0025 and *P* = 0.0089, respectively, Figure 3(b)), and HSPA2 was positively related to PSEN1 (*P* < 0.0001, Figure 3(b)). Moreover, the results also showed that HSPA1A and HSPA2 were negatively related to PSEN2 (*P* < 0.0001 and *P* = 0.0004, respectively, Figure 3(b)), and HSPA1A was negatively related to APOE (*P* = 0.0421, Figure 3(b)). In general, AD symptoms are associated with memory and cognition impairment. It has been suggested that cognitive deficit was associated with enhanced acetylcholinesterase activity in both cerebral cortex and hippocampus [10]. In view of this, we performed the correlation analysis between acetyl choline esterase (ACHE) and the 3 hub genes of HSP70 family in GSE122063 dataset. The results showed that HSPA2 was negatively related to ACHE (*P* < 0.0001, Figure 3(b)), and HSPA8 was positively related to ACHE (*P* = 0.0103, Figure 3(b)), but HSPA1A had no significant correlation (*P* = 0.2286, Figure 3(b)). Amyloid pathogenesis of AD reportedly starts with altered cleavage of APP by *β*-secretase and *γ*-secretase to produce insoluble A*β* fibrils [1, 43]. It suggests that *β*-secretase and *γ*-secretase play important roles in aggregates of A*β* plaques. Therefore, the correlation was also analyzed between the hub genes and the secretases. The results showed that HSPA2 was positively related to *β*-secretase activity (*P* = 0.0058, Figure 3(c)), and HSPA8 were negatively related to *β*-secretase activity (*P* < 0.0001, Figure 3(c)). Moreover, HSPA8 was negatively related to *γ*-secretase activity (*P* = 0.0117, Figure 3(c)).

### 3.3. Immune-Related Analysis Indicates the Relevance of Immune Cells and Hub Genes to AD

**Figure 4:**
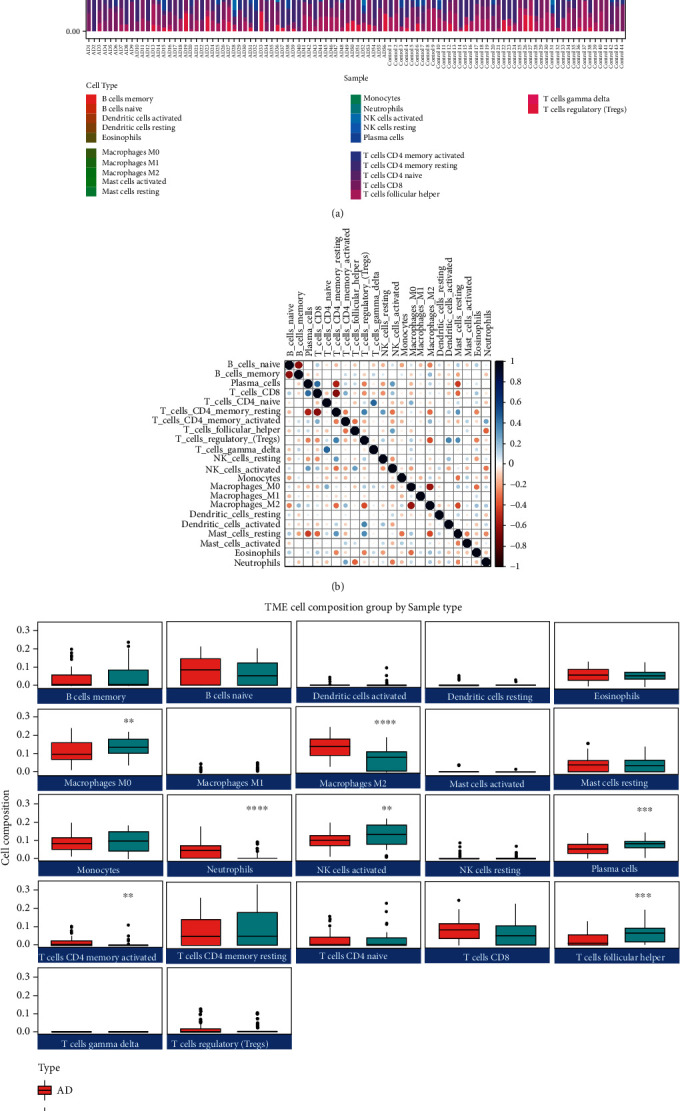
Immune-related analysis indicates the relevance of immune cells and hub genes to AD. (a) The proportion of the 22 immune cell types in AD and control samples in GSE122063 (AD: 56, control: 44). The horizontal coordinates indicate the samples, and the vertical coordinates indicate the proportion of immune cells. (b) Correlation matrix between the 22 immune cell types in GSE122063. Each row (column) represents one immune cell type. Blue represents positive correlations and red represents negative correlations. Darker colors indicate stronger correlations. (c) The total distribution of immune cells between the AD and control groups in GSE122063. The green box indicates the control group, and the red box indicates the AD group. Data were analyzed by Student's *t*-test and expressed as the Mean ± SD (AD: 56, control: 44). ∗∗ (*P* < 0.01), ∗∗∗ (*P* < 0.001), and ∗∗∗∗ (*P* < 0.0001) represent significance compared to the control group.

To investigate the relationship between 3 hub genes of immune-related HSP70 family in AD and the diverse immune infiltrating cells, we focused on the correlations between hub genes and immune cell type markers of various immune cells in AD samples of GSE122063. We analyzed the correlations between the expression of 3 hub genes and immune marker genes of different immune cells, such as B cells, neutrophils, and monocyte/macrophages. After the correlation analysis, the results suggested that there was a significant correlation between the expression of 3 hub genes and most immune markers (Table 2). We analyzed the immune infiltration of AD and control samples in GSE122063 by CIBERSORT algorithm.  Figure 4(a) shows that macrophages account for most significant proportion among the 22 immune cells in the samples. As shown in Figures 4(b) and 4(t), cells CD4 naive had the strongest positive correlation with T cells gamma delta (*r* = 0.46); nevertheless, T cells CD4 memory resting had the strongest negative correlation with T cells CD8 (*r* = −0.64). We also carried out immune cell composition analysis to further compare the difference in the immune cells between the AD and control groups in GSE122063. The results suggested that the infiltration of macrophages M2, neutrophils, and T cells CD4 memory activated was higher in AD than in control group (*P* < 0.0001, *P* < 0.0001, and *P* < 0.01, respectively, Figure 4(c)), while the infiltration of macrophages M0, NK cells activated, plasma cells, and T cells follicular helper was opposite (*P* < 0.01, *P* < 0.01, *P* < 0.001, and *P* < 0.001, respectively, Figure 4(c)). These data indicated that the distribution of seven immune cell types was related to the occurrence of AD.

### 3.4. Functional Enrichment Analysis of Hub Genes, Interaction Genes, and Star Molecules in AD

**Figure 5:**
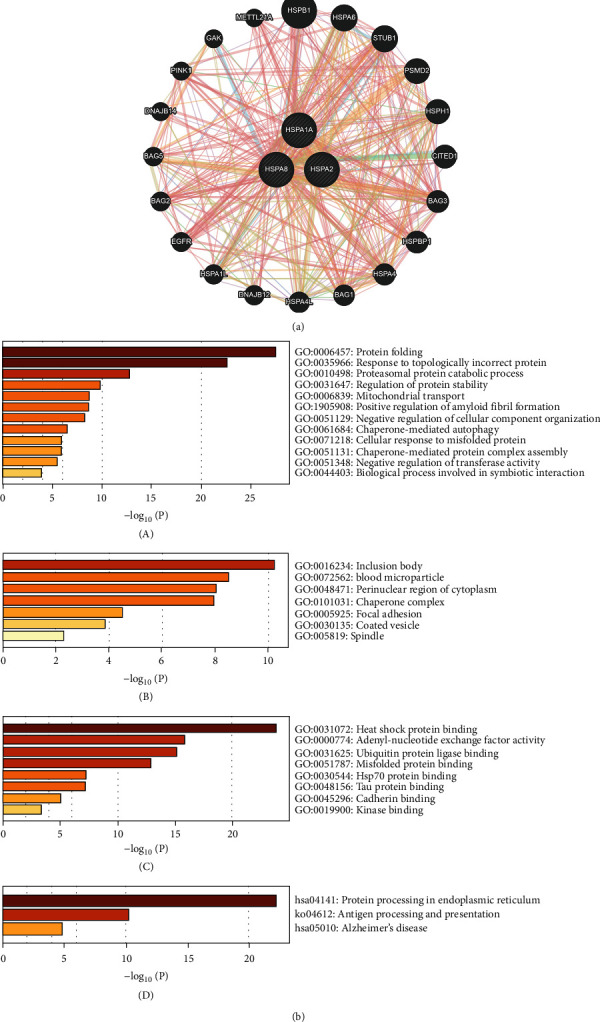
Functional enrichment analysis of hub genes, interaction genes, and star molecules in AD. (a) Prediction of interaction proteins with the 3 hub genes (HSPA1A, HSPA2, and HSPA8) of immune-related HSP70 family in AD are shown by protein–protein interaction network. (b) The GO and KEGG enrichment analysis of the 3 hub genes (HSPA1A, HSPA2, and HSPA8), 20 interaction genes (HSPB1, HSPA6, STUB1, PSMD2, HSPH1, CITED1, BAG3, HSPBP1, HSPA4, BAG1, HSPA4L, DNAJB12, HSPA1L, EGFR, BAG2, BAG5, DNAJB14, PINK1, GAK, and METTL21A), and 4 star molecules (APP, PSEN1, PSEN2, and APOE) in AD by Metascape. Bar plot of GO enrichment in biological process terms (A), cellular component terms (B), and molecular function terms (C). Bar plot of KEGG enriched terms (D).

Prediction of interaction proteins with the 3 hub genes of immune-related HSP70 family in AD was performed to explore the potential interactions using GeneMANIA. The results revealed that there were primarily 20 genes (*HSPB1*, *HSPA6*, *STUB1*, *PSMD2*, *HSPH1*, *CITED1*, *BAG3*, *HSPBP1*, *HSPA4*, *BAG1*, *HSPA4L*, *DNAJB12*, *HSPA1L*, *EGFR*, *BAG2*, *BAG5*, *DNAJB14*, *PINK1*, *GAK*, and *METTL21A*) associated with the 3 hub genes (Figure 5(a)). Then, the 3 hub genes, these interaction genes and star molecules in AD (APP, PSEN1, PSEN2, and APOE) were submitted in Metascape for analyzing the functions.  Figure 5(b) shows the most highly enriched GO items. In the biological process (BP) category, protein folding, response to topologically incorrect protein, proteasomal protein catabolic process, regulation of protein stability, mitochondrial transport, response to oxidative stress, and positive regulation of amyloid fibril formation were associated with the occurrence and progression of AD. In the cellular component (CC) category, the inclusion body, blood microparticle, perinuclear region of cytoplasm, chaperone complex, focal adhesion, coated vesicle, and spindle were the most highly enriched items. These genes were mainly enriched in heat shock protein binding, adenyl-nucleotide exchange factor activity, ubiquitin protein ligase binding, misfolded protein binding, HSP70 protein binding, Tau protein binding, and other aspects in the molecular function (MF) category. Additionally, KEGG pathway analyses showed that protein processing in endoplasmic reticulum, antigen processing, and presentation and Alzheimer's disease were significantly implicated in the occurrence and progression of AD (Figure 5(b)).

### 3.5. Construction of TF-Hub Gene-miRNA Network May Be a New Potential Regulatory Network of AD

**Figure 6:**
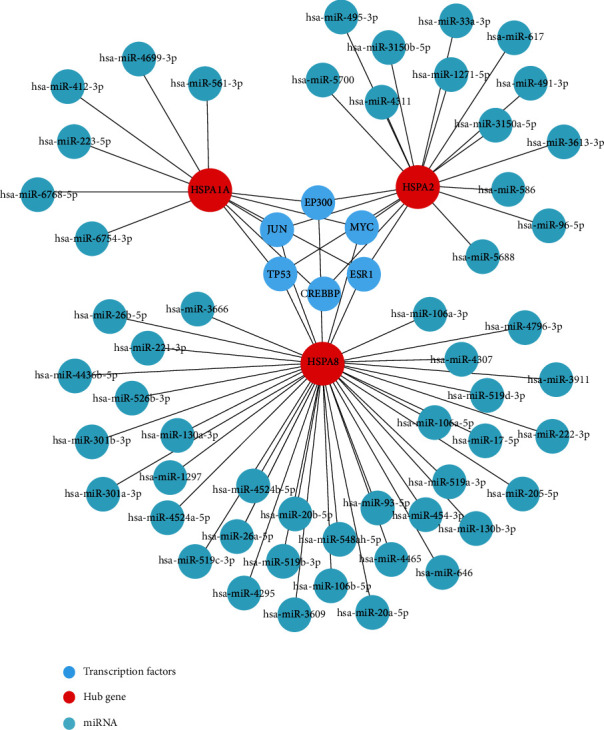
Construction of TF-hub gene-miRNA network may be a new potential regulatory network of AD. The red circle indicates the hub genes, the blue circle indicates the TF targets, and the brilliant blue circle indicates the miRNA targets.

Due to the significantly different expression levels of the 3 hub genes of immune-related HSP70 family in the AD vs. control groups, the feasible transcription factor (TF) targets of the hub genes were explored by hTFtarget. Then, the intersection of predicted TF was taken using the Jvenn website, and 130 possible TFs that jointly regulated the 3 hub genes were obtained (Supplementary Table 2). CytoHubba plug-in was further used to select the TFs with high scores in Cytoscape focusing screening of 6 algorithms (MCC, degree, MNC, closeness, radiality, and EPC) and taken intersection, and the last 6 important TF targets (EP300, MYC, TP53, JUN, CREBBP, and ESR1) of hub genes were obtained. Subsequently, miRNA targets of the hub genes were predicted using the two online databases, TargetScan and miRDB. The miRNAs predicted by these two programs overlapped were identified as miRNA targets, in which the miRNA score predicted by the miRDB was ≥80, and we obtained 6 miRNA targets of HSPA1A, 13 miRNA targets of HSPA2, and 35 miRNA targets of HSPA8, respectively (Supplementary Table 3). Finally, the TF-hub gene-miRNA network was constructed using the Cytoscape software (Figure 6). It may be a new potential regulatory network of AD.

## 4. Discussion

In this study, 3 hub genes of HSP70 family associated with AD were identified by computational analysis using gene expression data extracted from the GEO datasets. To our knowledge, this is the first report to screen HSP70 family members for identifying a series of hub genes as candidate biomarkers or therapeutic targets for AD and integrate clinical-related analysis, immune-related analysis, and biological function analysis of hub genes. According to ROC and correlation analyses, the 3 hub genes revealed potential clinical values. Moreover, immune cell infiltration analysis indicated the relevance of immune cells and hub genes to AD. Furthermore, our data suggested that EP300, MYC, TP53, JUN, CREBBP, and ESR1 might be key transcription factors (TFs) for the 3 hub genes and provided a new potential regulatory network of AD. Overall, we identified that HSPA1A, HSPA2, and HSPA8 among HSP70 family played vital roles in AD pathogenesis, and it provided a new perspective for AD diagnosis and treatment targets for AD. HSPA1A is a major heat shock protein in HSP70 family. A proteomics study of extracellular vesicles separated from cerebrospinal fluid (CSF) of AD showed that the expression level of HSPA1A was higher in the AD group than in the MCI and CTRL groups, and it might be used to monitor the progression of MCI to AD [44]. Another exon microarray data demonstrated that HSPA1A was found to be upregulated in patients with AD [23]. Our results showed increased expression of HSPA1A in AD, which is consistent with previous studies. This indicates HSPA1A may play an important part in AD progression. It was reported that HSPA2 was not only significantly highly expressed in late-onset Alzheimer's disease (LOAD), but also further elevated A*β*40 and A*β*42 levels and phosphorylated-tau in two cell lines with HSPA2 overexpression [24]. In our study, we found that HSPA2 was significantly overexpressed in AD. In addition, we also found that HSPA2 was significantly upregulated in Braak 5-6 when compared with Braak 0 in GSE131617. It is similar to the results of previous studies. This work further demonstrates HSPA2 as a specific key regulator of late-onset AD. In addition, HSPA8 was significantly downregulated in Braak 5-6 when compared with Braak 0 in our analysis. However, the expression level of HSPA1A decreased with different processes of AD. This suggests that the hub genes may not be a good predictor of the changes in different stages of AD, but limited by the dataset, no good research results could be obtained temporarily. Results of qRT-PCR revealed that a significant downregulation of HSPA8 was observed in AD across the three brain regions (entorhinal and auditory cortices and hippocampus) compared to the controls [45]. In this study, we found that HSPA8 decreased significantly in AD compared with control group in GSE5281. The results are consistent with previous studies. These findings indicate that HSPA1A, HSPA2, and HSPA8 play significant roles in the pathogenesis of AD.

A study revealed that the ROC result of HSPA1A mRNA in the diagnosis of myasthenia gravis was 0.830 [46]. Zhang et al. confirmed that HSPA8 as a potential serum biomarker for distinguishing renal cell carcinoma from benign urologic disease with ROC area under the curve (AUC) of 0.86 [47]. However, there is no assessment of the diagnostic ability of the hub genes in AD. Interestingly, we found that the combination of HSPA1A, HSPA2, and HSPA8 had a good ability to differentiate the AD and control groups in GSE122063. The AUC for predicting AD and control samples is 0.905 [95%confidence interval (CI) = 0.847–0.963]. The AUC of HSPA1A, HSPA2, and HSPA8 alone were 0.858 [95%confidence interval (CI) = 0.788–0.928], 0.821 [95%confidence interval (CI) = 0.739–0.902], and 0.667 [95%confidence interval (CI) = 0.562–0.772], respectively. This indicates that the predictive value of a single target is limited, while the combined target of the 3 hub genes has a higher predictive value.

Studies have shown that AD is a complex disorder with a relatively clear genetic component, involving several common AD-related genes. PSEN1, PSEN2, and APP have been identified as the cause of early onset familial AD (EOAD) [48]. In fact, early-onset autosomal dominant disease with age of onset younger than sixty years seems to be completely explained by pathogenic mutations in these three genes [49]. In LOAD, APOE is linked to the occurrence of AD and known to act in a dose-dependent manner. For instance, the effect of the *ε*4 allele in the risk for AD increases from 20 to 90% [50]. It was reported that the heat shock protein HSPA1A negatively regulated APP processing and A*β* production [51]. Our results showed that HSPA1A was negatively associated with APP. This demonstrated that HSPA1A was involved in the regulation mechanism of AD. In addition, a genetic association case-control study found that cross-talk in AD might be associated with multiway interactions between polymorphisms of candidate genes (HSPA1A, APOE, and so on) [52]. The correlation analysis in our study suggested that HSPA1A was negatively associated with APOE, further indicating a relationship between HSPA1A and susceptibility genes in AD. Interestingly, we also found that HSPA8 were negatively associated with APP, HSPA2 was positively associated with PSEN1, and HSPA1A and HSPA2 were negatively associated with PSEN2. These results have not been reported before. Moreover, AD causes varying degrees of memory and cognition impairment. Chonpathompikunlert et al. pointed out that acetyl choline esterase activity had played a role in the protection of neurodegeneration and improvement in memory impairment of AD's rats [53]. The results showed that HSPA2 was negatively related to ACHE and HSPA8 was positively related to ACHE. However, we have not found the appropriate dataset of GEO database which contains the level of acetyl choline to analyze the correlation with the 3 hub genes of HSP70 family. This may be due to the fact that the dataset related to AD in GEO database itself involves very few datasets of acetyl choline.

The main pathological process of AD is the accumulation of A*β* pathologic form which was caused by the continuous cleavage of APP via *β*- and *γ*-secretase in the brain [54]. Jo et al. reported that levels of *β*- and *γ*-secretase activities were greater in brain tissue samples from AD patients compared to nondemented control subjects [55]. It has been suggested that inhibition of the *β*- and *γ*-secretase was a valuable approach, which had been extensively tested in the clinic to prevent or delay the pathogenic effects of AD [56]. Based on the important role of *β*- and *γ*-secretase in A*β* formation, we analyzed the correlation in GSE106241 between the hub genes and *β*/*γ*-secretase activity, respectively. The results showed that HSPA2 was positively associated with *β*-secretase activity, HSPA8 was negatively associated with *β*-secretase activity, and HSPA8 was negatively associated with *γ*-secretase activity. However, due to the lack of HSPA1A data in this dataset, we did not confirm the correlation between HSPA1A and *β*- and *γ*-secretase activities and will add data later when available. Clinically, these results indicated that the 3 hub genes were closely relevant to the occurrence and development of AD. It may be possible to inhibit the activity of secretases by targeting the 3 hub genes, which in turn may have a therapeutic effect on AD.

There is evidence that immune system dysfunction in AD may be a major factor [57]. Many kinds of immune cells make up the immune system, including neutrophils and macrophages, to protect the body from external invasion. Hyperactivation of neutrophils is regarded as a characteristic of AD. The neutrophil formyl peptide receptors (FPR1 and FPR2) have become a therapeutic target that may be available for reducing injuries in inflammatory diseases including AD [58]. In our study, we found that HSPA1A and HSPA8 were significantly positive correlation with FPR1. Moreover, the infiltration of neutrophils in AD was significantly higher than in the control group. This suggests that these genes may play a part in neutrophils of immune system. It is worth mentioning that microglia had previously been reported to be associated with AD neuropathology and play a role in the etiology [59]. Microglia, as tissue-resident macrophages, play a fundamental role in the health of neurons [60]. Furthermore, the innate immune response may be modulated via polarizing microglia/macrophages in APP/PS1 mice [61]. In large genetic screens, key microglia/macrophages genes, such as CD33 and MS4As, have been associated with an increased risk of AD [59]. In this study, we found that HSPA1A, HSPA2, and HSPA8 were significantly correlated with CD33/MS4A4A through correlation analysis with immune cell type markers. In addition, the 3 hub genes have different levels of correlation with CD68, CD84, and other macrophages markers. The macrophages account for most significant proportion among the 22 immune cells in immune cell infiltration analysis, and we also found that infiltration of macrophages M2 was significantly increased in AD samples, while macrophages M0 decreased. These evidences may suggest that inflammatory activity of microglia in AD is increased and activated to promote the proliferation. This view is consistent with the research of Olmos-Alonso et al. [62]. NK cells have also been involved in AD [63], but controversial results have been published regarding NK cell number in AD patients. A study found no differences in the number of CD56 + CD16+ NK cells in AD patients [64], yet a decreased cells percentage of NK cells from 3xTg AD mice compared to NTg was reported by Maté et al. [65]. Our results are consistent with Ianire Maté's, that is, the infiltration of NK cells activated are reduced in AD, which may be due to the different functions of NK cells in different stages of AD. In general, our findings demonstrate an underlying immune role for HSPA1A/HSPA2/HSPA8 in AD.

The results of functional enrichment analysis indicated that 3 hub genes and their adjacent interaction genes mainly participated in biological process, such as protein folding, response to topologically incorrect protein, proteasomal protein catabolic process, regulation of protein stability, mitochondrial transport, response to oxidative stress, and positive regulation of amyloid fibril formation. The accumulation of misfolded protein aggregates is a common characteristic of AD, and it mainly includes an extensive aggregation of A*β*peptides and NFTs of hyper-phosphorylated tau protein [66]. There is the evidence that A*β* can be degraded through the proteasome to prevent its aggregation [67]. Several evidences suggest that the upregulation of molecular chaperones may inhibit NTFs' formation via dividing tau into productive folding pathways, thus preventing tau aggregation [68]. Moreover, a distinguishing feature of detrimental effects of A*β* peptides on neurons in AD is a significant decrease in mitochondria density and accumulation of mtDNA [69]. This may be due to an increase in mitochondrial degradation products by abnormal mitochondrial transport function, autophagy, or messed up proteolytic systems [70]. On the side, the enriched KEGG pathways include protein processing in endoplasmic reticulum (ER), antigen processing, and presentation and Alzheimer's disease. The ER is responsible for the proper folding or processing of nascent proteins, and the alterations in its homeostasis might be connected with accumulation of misfolded proteins in the brain of AD patients [71]. In addition, the analysis of TF-hub gene-miRNA network manifested that the 3 hub genes were related to TF targets EP300, MYC, TP53, JUN, CREBBP, and ESR1. Among them, it has been reported that MYC was increased in vulnerable neurons of AD patients, and the increased expression level of MYC might be in connection with cell death of neurons and the regulation of AD-related genes [72, 73]. An integrative analysis of hippocampus gene expression profiles also found dysregulation of transcription factors (CEBPB and MYC) in AD [74]. The miRNAs, a class of small noncoding RNAs (20-22 nucleotides), regulate the expression of key proteins and alter the expression of some miRNAs in AD pathogenesis. In our study, HSPA1A, HSPA2, and HSPA8 were predicted to be regulated by 6 miRNA targets, 13 miRNA targets, and 35 miRNA targets, respectively. It has been shown that hsa-miR-26b-5p can regulate the expression of the transporter vGLUT2, and a possible increased vGLUT2 level may lead to a higher intake of glutamate in the presynaptic vesicles to protect the nervous system from the excitatory toxicity of the glutamate itself, improving memory and cognition in AD [75]. We found that HSPA8 may be regulated by hsa-miR-26b-5p and then participate in the regulation of AD. However, studies on the regulation of miRNAs of HSP70 family members in AD are rare at present. Therefore, our findings may provide a new perspective to explore novel regulatory mechanism for research in this area.

The present study has some limitations. First, based on GEO datasets, some genes were lost due to inappropriate matching. The correlation analysis was lacking between the hub genes of HSP70 family and the definite AD biomarker including A*β*42, phospho-tau, and total tau due to the fact that the datasets in GEO database related to these AD biomarkers were very few. Second, the analysis of expression profile cannot reflect global changes, but only changes in transcription level. Third, further independent cohort with a larger sample size and molecular biological experiments are required to verify the roles of these hub genes in AD pathogenesis. To sum up, we hope our findings provide novel insights of AD pathogenesis and help to provide a set of potential helpful targets for further research on molecular mechanism and biomarkers in future.

## 5. Conclusion

We first reported that screens HSP70 family members to identify a series of hub genes in AD and performs clinical-related analysis, immune-related analysis, and biological function analysis of hub genes. Thus, HSPA1A, HSPA2, and HSPA8 may be 3 hub genes of immune-related HSP70 family in AD. Our findings might provide a novel understanding of AD pathogenesis and provide a set of potential useful targets for further studies on molecular mechanism and biomarkers.

## Figures and Tables

**Table 1:** The 17 members of the HSP70 family in human.

Gene	Gene ID	UniProt ID
*HSPA1A*	3303	P0DMV8
*HSPA1B*	3304	P0DMV9
*HSPA1L*	3305	P34931
*HSPA2*	3306	P54652
*HSPA4*	3308	P34932
*HSPA4L*	22824	O95757
*HSPA5*	3309	P11021
*HSPA6*	3310	P17066
*HSPA7*	3311	P48741
*HSPA8*	3312	P11142
*HSPA9*	3313	P38646
*HSPA12A*	259217	O43301
*HSPA12B*	116835	Q96MM6
*HSPA13*	6782	P48723
*HSPA14*	51182	Q0VDF9
*HSPH1*	10808	Q92598
*HYOU1*	10525	Q9Y4L1

**Table 2:** Correlation analysis between HSPA1A/HSPA2/HSPA8 and immune cell type markers in AD samples of GSE122063.

Cell type	Gene markers	HSPA1A		HSPA2		HSPA8	
		COR	*P* value	COR	*P* value	COR	*P* value
CD8+ T cells	CD8A	-0.2879	0.0314	-0.5776	<0.0001	0.0861	0.5280
Th1 cells	TBX21	0.0174	0.8985	0.3440	0.0094	-0.3606	0.0063
Treg	FOXP3	-0.2967	0.0264	0.1456	0.2843	-0.3557	0.0071
	CCR8	0.1485	0.2746	0.1529	0.2607	-0.0801	0.5573
B cells	CD22	0.0331	0.8087	0.6700	< 0.0001	-0.2869	0.0321
Neutrophils	CEACAM3	0.1321	0.3319	0.3057	0.0220	0.0054	0.9685
	FPR1	0.5884	<0.0001	-0.1791	0.1866	0.4027	0.0021
	CSF3R	-0.0662	0.6280	0.0049	0.9713	-0.1613	0.2349
	S100A12	0.1714	0.2065	0.3556	0.0072	-0.2007	0.1381
Monocyte/macrophages	CD86	-0.0057	0.9667	0.4965	< 0.0001	-0.2304	0.0876
	CSF1R	0.2432	0.0709	0.01097	0.9360	0.1407	0.3009
	CD68	0.4656	0.0003	-0.0192	0.8883	0.3343	0.0118
	CD84	0.3693	0.0051	-0.0025	0.9852	0.3444	0.0093
	CD163	0.3700	0.0050	0.1468	0.2803	0.0874	0.5221
	MS4A4A	0.4919	0.0001	-0.3283	0.0135	0.4726	0.0002
	CD14	0.4918	0.0001	-0.3242	0.0148	0.3908	0.0029
	CD33	0.2943	0.0277	-0.3928	0.0028	0.5218	< 0.0001
NK cells	KIR3DL3	-0.1883	0.1646	0.1963	0.1470	-0.3442	0.0094
Dendritic cells	CD83	-0.1420	0.2964	-0.5224	< 0.0001	0.1111	0.4151

## Data Availability

The authors declare that all relevant data of this study are available within the article or from the corresponding author on reasonable request.

## Supplementary Materials

Supplementary Materials Supplementary Fig.1 Expression of three immune-related HSP70 family members in GSE132903. The blue box indicates the control group, and the orange box indicates the AD group. Data were analyzed by Student's T-test and expressed as the Mean ± SD. ∗*P* < 0.05; ∗∗∗*P* < 0.001. Supplementary Table 1. Immune molecules in the Immport database. Supplementary Table 2. Common TFs of the 3 hub genes from hTFtarget by jvenn. Supplementary Table 3. The overlapped miRNAs of HSPA1A/HSPA2/HSPA8 predicted by TargetScan and miRDB
