# Supplementary material for: HSPA1A, HSPA2, and HSPA8 Are Potential Molecular Biomarkers for Prognosis among HSP70 Family in Alzheimer's Disease
Source: Dis Markers. 2022 Sep 30;2022:9480398. doi: 10.1155/2022/9480398 (PMC9553556; doi:10.1155/2022/9480398)
Supplement: Supplementary Materials — Supplementary Fig.1 Expression of three immune-related HSP70 family members in GSE132903. The blue box indicates the control group, and the orange box indicates the AD group. Data were analyzed by Student's T-test and expressed as the Mean ± SD. ∗P < 0.05; ∗∗∗P < 0.001. Supplementary Table 1. Immune molecules in the Immport database. Supplementary Table 2. Common TFs of the 3 hub genes from hTFtarget by jvenn. Supplementary Table 3. The overlapped miRNAs of HSPA1A/HSPA2/HSPA8 predicted by TargetScan and miRDB [file 9480398.f1.zip › Supplementary Table2-0324.docx]

**Supplementary Table 2. Common TFs of the 3 hub genes from hTFtarget by jvenn**

| **No. of datasets** | **TFs** | **Tissue** | **The peak close to TSS** | **The peak with strongest signal** |
| --- | --- | --- | --- | --- |
| 1 | TAF3 | Colon | Chr6,31806417,31807167,18.0,-904,pr,dataset-3480 | Chr6,31806417,31807167,18.0,-904,pr,dataset-3480 |
| 3 | STAT1 | Blood | Chr6,31815474,31815716,3.18,10,pr,dataset-3392 | Chr6,31814610,31814969,6.10,-854,pr,dataset-3391 |
| 1 | ESR1 | Bone | Chr6,31810397,31810875,26.9,-506,pr,dataset-4080 | Chr6,31810397,31810875,26.9,-506,pr,dataset-4080 |
| 20 | FOXA1 | Breast | Chr6,31815502,31815624,5.06,38,pr,dataset-1272 | Chr6,31814515,31815381,27.3,-949,pr,dataset-4838 |
| 1 | ETV1 | Prostate | Chr6,31815523,31815750,16.6,59,pr,dataset-1169 | Chr6,31815523,31815750,16.6,59,pr,dataset-1169 |
| 2 | CEBPB | Blood | Chr6,31817605,31817807,5.24,2141,gb,dataset-5019 | Chr6,31806665,31807027,9.92,-879,pr,dataset-5019 |
| 3 | RUNX1 | Blood | Chr6,31815504,31815743,8.82,40,pr,dataset-2830 | Chr6,31806667,31807095,26.1,-879,pr,dataset-2829 |
| 1 | ZNF263 | Bone marrow | Chr6,31806718,31807038,4.61,-874,pr,dataset-3832 | Chr6,31806718,31807038,4.61,-874,pr,dataset-3832 |
| 1 | NFYB | Blood | Chr6,31815229,31815613,22.5,-235,pr,dataset-2263 | Chr6,31815229,31815613,22.5,-235,pr,dataset-2263 |
| 2 | CBFB | Blood | Chr6,31806671,31807074,24.9,-879,pr,dataset-331 | Chr6,31806671,31807074,24.9,-879,pr,dataset-331 |
| 1 | CEBPA | Blood | Chr6,31806775,31807016,5.31,-868,pr,dataset-5018 | Chr6,31806775,31807016,5.31,-868,pr,dataset-5018 |
| 1 | KDM4C | Oesophagus | Chr6,31815336,31815703,4.90,-128,pr,dataset-1863 | Chr6,31806251,31807081,19.6,-921,pr,dataset-1863 |
| 1 | RBBP5 | Bone marrow | Chr6,31815159,31815835,19.4,-305,pr,dataset-2660 | Chr6,31815159,31815835,19.4,-305,pr,dataset-2660 |
| 2 | CDK9 | Blood | Chr6,31814631,31814929,4.99,-833,pr,dataset-386 | Chr6,31806590,31807236,9.87,-887,pr,dataset-387 |
| 1 | TAF7 | Embryo | Chr6,31815527,31815661,3.39,63,pr,dataset-3481 | Chr6,31806797,31806911,5.14,-866,pr,dataset-3481 |
| 1 | SP4 | Embryo | Chr6,31815244,31815655,5.07,-220,pr,dataset-3078 | Chr6,31806710,31807136,5.66,-875,pr,dataset-3078 |
| 8 | TFAP2C | Breast | Chr6,31815446,31815722,19.0,-18,pr,dataset-3600 | Chr6,31806641,31807020,24.7,-882,pr,dataset-3599 |
| 1 | HIF1A | Breast | Chr6,31814572,31815784,15.8,-892,pr,dataset-1634 | Chr6,31814572,31815784,15.8,-892,pr,dataset-1634 |
| 1 | MAZ | Blood | Chr6,31806561,31807096,15.4,-890,pr,dataset-4615 | Chr6,31806561,31807096,18.9,-890,pr,dataset-4615 |
| 1 | NRF1 | Bone marrow | Chr6,31806785,31807167,16.6,-867,pr,dataset-2360 | Chr6,31806785,31807167,16.6,-867,pr,dataset-2360 |
| 3 | SMARCA4 | Other | Chr6,31807149,31807511,5.64,-831,pr,dataset-2991 | Chr6,31806361,31806919,7.35,-910,pr,dataset-2990 |
| 1 | MXI1 | Bone marrow | Chr6,31815473,31815753,4.58,9,pr,dataset-2065 | Chr6,31806573,31806919,5.23,-889,pr,dataset-2065 |
| 3 | E2F6 | Bone marrow | Chr6,31815447,31815615,3.99,-17,pr,dataset-822 | Chr6,31806657,31807130,18.8,-880,pr,dataset-820 |
| 2 | FOS | Bone marrow | Chr6,31815294,31815586,14.1,-170,pr,dataset-1244 | Chr6,31815292,31815603,17.6,-172,pr,dataset-1255 |
| 1 | WDR5 | Keratinocyte | Chr6,31814989,31815249,3.61,-475,pr,dataset-3735 | Chr6,31814989,31815249,3.61,-475,pr,dataset-3735 |
| 1 | BRD2 | Kidney | Chr6,31815537,31815784,7.48,73,pr,dataset-253 | Chr6,31813821,31815428,28.8,-164,pr,dataset-253 |
| 3 | TCF12 | Blood | Chr6,31815484,31815674,19.1,20,pr,dataset-3525 | Chr6,31806684,31807059,26.6,-878,pr,dataset-3533 |
| 1 | HSF1 | Bone marrow | Chr6,31815199,31815691,13.8,-265,pr,dataset-1712 | Chr6,31815199,31815691,13.8,-265,pr,dataset-1712 |
| 1 | E2F4 | Bone marrow | Chr6,31815442,31815803,7.71,-22,pr,dataset-810 | Chr6,31806676,31807150,12.3,-878,pr,dataset-810 |
| 2 | MED1 | Blood | Chr6,31815457,31815646,4.55,-7,pr,dataset-2003 | Chr6,31806466,31807219,11.8,-899,pr,dataset-2003 |
| 1 | GATA1 | Bone marrow | Chr6,31815602,31815764,5.26,138,pr,dataset-1454 | Chr6,31806130,31806940,11.5,-933,pr,dataset-1454 |
| 6 | EP300 | Blood | Chr6,31815446,31815740,6.02,-18,pr,dataset-929 | Chr6,31806647,31807138,11.4,-881,pr,dataset-4504 |
| 1 | BHLHE40 | Bone marrow | Chr6,31814689,31814930,4.82,-775,pr,dataset-233 | Chr6,31806679,31806997,4.85,-878,pr,dataset-233 |
| 2 | ELF1 | Blood | Chr6,31815517,31815742,4.52,53,pr,dataset-855 | Chr6,31806651,31807095,22.6,-881,pr,dataset-863 |
| 1 | STAT5B | Blood | Chr6,31817592,31817821,7.97,2128,gb,dataset-3434 | Chr6,31806903,31807092,7.98,-856,pr,dataset-3434 |
| 1 | EGR1 | Blood | Chr6,31814579,31814847,6.09,-885,pr,dataset-840 | Chr6,31814579,31814847,6.09,-885,pr,dataset-840 |
| 1 | LMNB1 | Lung | Chr6,31814890,31815214,2.71,-574,pr,dataset-1931 | Chr6,31813320,31813674,4.20,-214,pr,dataset-1931 |
| 1 | KDM5B | Bone marrow | Chr6,31815174,31815820,17.0,-290,pr,dataset-1869 | Chr6,31815174,31815820,17.0,-290,pr,dataset-1869 |
| 2 | BRD3 | Blood | Chr6,31806702,31807199,11.5,-876,pr,dataset-265 | Chr6,31806694,31807185,33.6,-877,pr,dataset-264 |
| 1 | FOXP1 | Embryo | Chr6,31815157,31815567,4.57,-307,pr,dataset-1400 | Chr6,31814658,31814869,7.84,-806,pr,dataset-1400 |
| 14 | POLR2A | Blood | Chr6,31815462,31815681,20.2,-2,pr,dataset-2491 | Chr6,31815125,31815772,72.5,-339,pr,dataset-2498 |
| 2 | HDAC1 | Blood | Chr6,31815497,31815693,4.69,33,pr,dataset-1587 | Chr6,31806496,31807168,28.4,-896,pr,dataset-4505 |
| 3 | FLI1 | Blood | Chr6,31815444,31815762,21.6,-20,pr,dataset-4499 | Chr6,31806391,31807115,103.,-907,pr,dataset-1229 |
| 3 | YY1 | Blood | Chr6,31815523,31815673,4.38,59,pr,dataset-3759 | Chr6,31814650,31814836,11.5,-814,pr,dataset-3761 |
| 1 | JUND | Bone marrow | Chr6,31815463,31815762,4.29,-1,pr,dataset-1828 | Chr6,31806610,31807178,10.0,-885,pr,dataset-1828 |
| 1 | SUZ12 | Liver | Chr6,31815289,31815763,6.16,-175,pr,dataset-3462 | Chr6,31806750,31807095,11.7,-871,pr,dataset-3462 |
| 1 | ARNT | Breast | Chr6,31815088,31815729,6.03,-376,pr,dataset-155 | Chr6,31814650,31814944,9.87,-814,pr,dataset-155 |
| 2 | MYB | Blood | Chr6,31806697,31806983,2.70,-876,pr,dataset-2076 | Chr6,31806677,31806927,5.03,-878,pr,dataset-2079 |
| 1 | RELA | Blood | Chr6,31814706,31814911,6.59,-758,pr,dataset-2692 | Chr6,31814706,31814911,6.59,-758,pr,dataset-2692 |
| 1 | MED12 | Colon | Chr6,31814545,31814927,4.82,-919,pr,dataset-2026 | Chr6,31806601,31807161,10.5,-886,pr,dataset-2026 |
| 1 | RBL2 | Lung | Chr6,31815242,31815501,6.20,-222,pr,dataset-2670 | Chr6,31815242,31815501,6.20,-222,pr,dataset-2670 |
| 2 | NFYA | Bone marrow | Chr6,31815270,31815616,8.25,-194,pr,dataset-2256 | Chr6,31815208,31815641,19.4,-256,pr,dataset-2261 |
| 1 | CREB1 | Bone marrow | Chr6,31815204,31815740,2.94,-260,pr,dataset-473 | Chr6,31806564,31807028,11.9,-890,pr,dataset-473 |
| 1 | CREBBP | Breast | Chr6,31806756,31806897,7.03,-870,pr,dataset-494 | Chr6,31806756,31806897,7.03,-870,pr,dataset-494 |
| 1 | TEAD4 | Bone marrow | Chr6,31815457,31815825,6.24,-7,pr,dataset-3571 | Chr6,31817514,31819287,10.3,2050,gb,dataset-3571 |
| 3 | SPI1 | Adrenal gland | Chr6,31814478,31815776,21.6,-986,pr,dataset-3155 | Chr6,31806630,31807255,41.3,-883,pr,dataset-3234 |
| 1 | GTF2B | Bone marrow | Chr6,31815380,31815693,12.4,-84,pr,dataset-1552 | Chr6,31815380,31815693,12.4,-84,pr,dataset-1552 |
| 1 | REST | Bone marrow | Chr6,31815206,31815790,11.4,-258,pr,dataset-2759 | Chr6,31814534,31815154,23.0,-930,pr,dataset-2759 |
| 1 | NCOR1 | Kidney | Chr6,31815183,31815790,5.76,-281,pr,dataset-2204 | Chr6,31806628,31807156,8.37,-883,pr,dataset-2204 |
| 3 | BCOR | Embryo | Chr6,31815487,31815845,44.0,23,pr,dataset-3990 | Chr6,31815487,31815845,44.0,23,pr,dataset-3990 |
| 1 | MYH11 | Blood | Chr6,31806689,31806977,18.2,-877,pr,dataset-2171 | Chr6,31806689,31806977,18.2,-877,pr,dataset-2171 |
| 1 | IRF1 | Blood | Chr6,31814588,31814885,6.31,-876,pr,dataset-1767 | Chr6,31814588,31814885,6.31,-876,pr,dataset-1767 |
| 3 | PAX5 | Blood | Chr6,31815506,31815655,5.04,42,pr,dataset-2399 | Chr6,31806653,31807012,9.89,-881,pr,dataset-2398 |
| 1 | ETS1 | Blood | Chr6,31806613,31807161,13.6,-885,pr,dataset-1164 | Chr6,31806613,31807161,13.6,-885,pr,dataset-1164 |
| 1 | THAP1 | Bone marrow | Chr6,31815536,31815672,3.15,72,pr,dataset-3611 | Chr6,31806696,31807058,6.24,-876,pr,dataset-3611 |
| 1 | SUMO2 | Kidney | Chr6,31806669,31806903,2.73,-879,pr,dataset-3445 | Chr6,31806669,31806903,2.73,-879,pr,dataset-3445 |
| 15 | STAG1 | Blood | Chr6,31817618,31817858,4.32,2154,gb,dataset-3377 | Chr6,31806481,31807161,28.7,-898,pr,dataset-3372 |
| 5 | FOXA2 | Embryo | Chr6,31815379,31815646,3.40,-85,pr,dataset-1360 | Chr6,31814860,31815212,8.43,-604,pr,dataset-1357 |
| 1 | SRF | Breast | Chr6,31814699,31814941,3.65,-765,pr,dataset-3345 | Chr6,31814699,31814941,3.65,-765,pr,dataset-3345 |
| 2 | RXRA | Colon | Chr6,31806621,31806736,4.33,-884,pr,dataset-2873 | Chr6,31806442,31807112,7.13,-902,pr,dataset-2869 |
| 1 | TP53 | Blood | Chr6,31806711,31806862,5.72,-875,pr,dataset-3631 | Chr6,31805971,31806174,8.25,-949,pr,dataset-3631 |
| 1 | USF1 | Brain | Chr6,31815163,31815396,5.60,-301,pr,dataset-3710 | Chr6,31815163,31815396,5.60,-301,pr,dataset-3710 |
| 1 | HDAC2 | Blood | Chr6,31815449,31815755,7.44,-15,pr,dataset-4506 | Chr6,31806561,31807105,26.1,-890,pr,dataset-4506 |
| 1 | ELK3 | Vein | Chr6,31806839,31807072,6.47,-862,pr,dataset-879 | Chr6,31806839,31807072,6.47,-862,pr,dataset-879 |
| 2 | E2F1 | Blood | Chr6,31806625,31807228,72.7,-883,pr,dataset-4737 | Chr6,31806625,31807228,72.7,-883,pr,dataset-4737 |
| 5 | ERG | Blood | Chr6,31815447,31815772,21.5,-17,pr,dataset-4500 | Chr6,31806645,31807148,29.9,-881,pr,dataset-986 |
| 1 | MAX | Blood | Chr6,31806644,31807039,3.57,-882,pr,dataset-1983 | Chr6,31806644,31807039,3.57,-882,pr,dataset-1983 |
| 1 | KLF9 | Other | Chr6,31814160,31815615,22.4,-130,pr,dataset-1904 | Chr6,31814160,31815615,22.4,-130,pr,dataset-1904 |
| 1 | PBX3 | Blood | Chr6,31815184,31815286,5.61,-280,pr,dataset-2413 | Chr6,31814669,31814794,12.1,-795,pr,dataset-2413 |
| 1 | SP1 | Blood | Chr6,31815282,31815624,7.52,-182,pr,dataset-3057 | Chr6,31806707,31807136,17.6,-875,pr,dataset-3057 |
| 1 | NR2F2 | Bone marrow | Chr6,31815442,31815771,3.35,-22,pr,dataset-2307 | Chr6,31815442,31815771,3.35,-22,pr,dataset-2307 |
| 1 | SIN3A | Brain | Chr6,31815473,31815797,9.72,9,pr,dataset-2907 | Chr6,31806623,31807223,10.9,-884,pr,dataset-2907 |
| 7 | BRD4 | Blood | Chr6,31814544,31814809,6.63,-920,pr,dataset-269 | Chr6,31806604,31807295,16.3,-886,pr,dataset-269 |
| 1 | HEY1 | Bone marrow | Chr6,31815434,31815766,19.1,-30,pr,dataset-1621 | Chr6,31815434,31815766,19.1,-30,pr,dataset-1621 |
| 4 | GABPA | Blood | Chr6,31815459,31815799,9.86,-5,pr,dataset-1434 | Chr6,31815506,31815836,12.8,42,pr,dataset-1435 |
| 1 | PPARGC1A | Other | Chr6,31815247,31815522,8.78,-217,pr,dataset-2585 | Chr6,31815247,31815522,8.78,-217,pr,dataset-2585 |
| 1 | CTCF | Aortic adventitial | Chr6,31806879,31806975,7.41,-858,pr,dataset-676 | Chr6,31806879,31806975,7.41,-858,pr,dataset-676 |
| 1 | FOXM1 | Blood | Chr6,31812627,31812836,3.36,-283,pr,dataset-1381 | Chr6,31812627,31812836,3.36,-283,pr,dataset-1381 |
| 2 | KLF5 | Colon | Chr6,31814675,31814891,6.86,-789,pr,dataset-1892 | Chr6,31814675,31814891,6.86,-789,pr,dataset-1892 |
| 3 | STAT3 | Breast | Chr6,31815498,31815641,4.90,34,pr,dataset-3402 | Chr6,31806643,31807260,46.3,-882,pr,dataset-3401 |
| 1 | CBX8 | Prostate | Chr6,31814208,31815936,36.7,-125,pr,dataset-351 | Chr6,31814208,31815936,36.7,-125,pr,dataset-351 |
| 1 | HCFC1 | Bone marrow | Chr6,31815522,31815755,2.70,58,pr,dataset-1581 | Chr6,31806556,31807196,12.1,-890,pr,dataset-1581 |
| 1 | MYC | Bone | Chr6,31815579,31815765,6.65,115,pr,dataset-2123 | Chr6,31806859,31806944,6.66,-860,pr,dataset-2123 |
| 1 | PGR | Breast | Chr6,31814716,31814984,8.31,-748,pr,dataset-2429 | Chr6,31814716,31814984,8.31,-748,pr,dataset-2429 |
| 2 | ELK4 | Cervix | Chr6,31815364,31815790,5.78,-100,pr,dataset-882 | Chr6,31815364,31815790,5.78,-100,pr,dataset-882 |
| 1 | TBL1XR1 | Bone marrow | Chr6,31814641,31814927,5.70,-823,pr,dataset-3512 | Chr6,31806627,31806994,6.49,-883,pr,dataset-3512 |
| 1 | RUNX3 | Blood | Chr6,31814739,31814885,2.64,-725,pr,dataset-2864 | Chr6,31812537,31812869,4.91,-292,pr,dataset-2864 |
| 1 | TFAP4 | Blood | Chr6,31815418,31815773,4.10,-46,pr,dataset-3604 | Chr6,31806923,31807338,5.43,-854,pr,dataset-3604 |
| 1 | ZBTB7A | Bone marrow | Chr6,31814853,31815753,12.7,-611,pr,dataset-3796 | Chr6,31814853,31815753,12.7,-611,pr,dataset-3796 |
| 1 | DDX5 | Other | Chr6,31814325,31814576,2.75,-113,pr,dataset-790 | Chr6,31807214,31807989,4.48,-825,pr,dataset-790 |
| 2 | PHF8 | Bone marrow | Chr6,31814737,31815851,47.2,-727,pr,dataset-2446 | Chr6,31814736,31815853,50.7,-728,pr,dataset-2440 |
| 1 | BCL11A | Blood | Chr6,31814695,31814893,5.04,-769,pr,dataset-209 | Chr6,31814695,31814893,5.04,-769,pr,dataset-209 |
| 2 | CDK7 | Blood | Chr6,31807031,31807206,3.87,-843,pr,dataset-376 | Chr6,31806951,31807221,4.51,-851,pr,dataset-377 |
| 1 | IRF4 | Blood | Chr6,31815469,31815645,4.39,5,pr,dataset-1778 | Chr6,31806663,31807023,6.68,-880,pr,dataset-1778 |
| 1 | NFIC | Blood | Chr6,31812616,31812875,5.20,-284,pr,dataset-2241 | Chr6,31812616,31812875,5.20,-284,pr,dataset-2241 |
| 1 | RAD21 | Blood | Chr6,31817735,31817951,17.2,2271,gb,dataset-2598 | Chr6,31817735,31817951,17.2,2271,gb,dataset-2598 |
| 1 | CBX3 | Bone marrow | Chr6,31815502,31815775,3.48,38,pr,dataset-340 | Chr6,31815502,31815775,3.48,38,pr,dataset-340 |
| 1 | HSF2 | Bone marrow | Chr6,31814660,31815776,47.2,-804,pr,dataset-1744 | Chr6,31814660,31815776,47.2,-804,pr,dataset-1744 |
| 1 | JUN | Bone marrow | Chr6,31815500,31815707,6.25,36,pr,dataset-1815 | Chr6,31806695,31807184,8.53,-876,pr,dataset-1815 |
| 1 | PRAME | Bone marrow | Chr6,31815317,31815636,4.40,-147,pr,dataset-2586 | Chr6,31815317,31815636,4.40,-147,pr,dataset-2586 |
| 1 | SP2 | Bone marrow | Chr6,31815305,31815528,4.13,-159,pr,dataset-3070 | Chr6,31815305,31815528,4.13,-159,pr,dataset-3070 |
| 1 | ZNF143 | Bone marrow | Chr6,31815515,31815624,2.69,51,pr,dataset-3814 | Chr6,31806842,31807103,5.13,-862,pr,dataset-3814 |
| 1 | RFX5 | Brain | Chr6,31815370,31815501,2.97,-94,pr,dataset-2813 | Chr6,31815370,31815501,2.97,-94,pr,dataset-2813 |
| 1 | EGLN2 | Breast | Chr6,31815456,31815721,5.51,-8,pr,dataset-838 | Chr6,31815456,31815721,5.51,-8,pr,dataset-838 |
| 2 | GRHL2 | Bronchial | Chr6,31807027,31807193,5.98,-843,pr,dataset-1550 | Chr6,31807027,31807193,5.98,-843,pr,dataset-1550 |
| 1 | BTAF1 | Cervix | Chr6,31815273,31815793,6.94,-191,pr,dataset-316 | Chr6,31815273,31815793,6.94,-191,pr,dataset-316 |
| 1 | SMC3 | Cervix | Chr6,31815577,31815712,2.24,113,pr,dataset-3007 | Chr6,31806680,31807152,4.71,-878,pr,dataset-3007 |
| 1 | CDK8 | Colon | Chr6,31814580,31814805,7.05,-884,pr,dataset-382 | Chr6,31814580,31814805,7.05,-884,pr,dataset-382 |
| 1 | E2F7 | Colon | Chr6,31806355,31806969,6.24,-910,pr,dataset-826 | Chr6,31806355,31806969,6.24,-910,pr,dataset-826 |
| 1 | SMC1A | Colon | Chr6,31814575,31814886,7.06,-889,pr,dataset-3004 | Chr6,31806576,31807177,12.5,-888,pr,dataset-3004 |
| 1 | VEZF1 | Colon | Chr6,31814579,31814869,5.35,-885,pr,dataset-3733 | Chr6,31806602,31806999,8.17,-886,pr,dataset-3733 |
| 1 | CTBP2 | Embryo | Chr6,31815296,31815707,4.34,-168,pr,dataset-500 | Chr6,31806682,31807031,5.88,-878,pr,dataset-500 |
| 1 | KLF4 | Foreskin | Chr6,31814716,31814868,6.50,-748,pr,dataset-1888 | Chr6,31814716,31814868,6.50,-748,pr,dataset-1888 |
| 1 | MAFB | Foreskin | Chr6,31815463,31815660,16.9,-1,pr,dataset-1944 | Chr6,31815463,31815660,16.9,-1,pr,dataset-1944 |
| 1 | KLF1 | Immortalized Human Erythroid Progenitor Cell Line | Chr6,31815551,31815760,4.62,87,pr,dataset-3923 | Chr6,31815551,31815760,4.62,87,pr,dataset-3923 |
| 1 | CEBPD | Liver | Chr6,31815452,31815680,8.98,-12,pr,dataset-438 | Chr6,31806602,31807089,10.7,-886,pr,dataset-438 |
| 1 | BCL3 | Lung | Chr6,31815436,31815727,8.73,-28,pr,dataset-215 | Chr6,31815436,31815727,8.73,-28,pr,dataset-215 |
| 2 | RB1 | Lung | Chr6,31815246,31815783,12.6,-218,pr,dataset-2653 | Chr6,31815246,31815783,12.6,-218,pr,dataset-2653 |
| 1 | ONECUT1 | Other | Chr6,31814643,31814983,33.4,-821,pr,dataset-2373 | Chr6,31814643,31814983,33.4,-821,pr,dataset-2373 |
| 1 | BCL6 | Tonsil | Chr6,31814582,31814876,5.33,-882,pr,dataset-224 | Chr6,31814582,31814876,5.33,-882,pr,dataset-224 |
