# Supplementary material for: HSPA1A, HSPA2, and HSPA8 Are Potential Molecular Biomarkers for Prognosis among HSP70 Family in Alzheimer's Disease
Source: Dis Markers. 2022 Sep 30;2022:9480398. doi: 10.1155/2022/9480398 (PMC9553556; doi:10.1155/2022/9480398)
Supplement: Supplementary Materials — Supplementary Fig.1 Expression of three immune-related HSP70 family members in GSE132903. The blue box indicates the control group, and the orange box indicates the AD group. Data were analyzed by Student's T-test and expressed as the Mean ± SD. ∗P < 0.05; ∗∗∗P < 0.001. Supplementary Table 1. Immune molecules in the Immport database. Supplementary Table 2. Common TFs of the 3 hub genes from hTFtarget by jvenn. Supplementary Table 3. The overlapped miRNAs of HSPA1A/HSPA2/HSPA8 predicted by TargetScan and miRDB [file 9480398.f1.zip › Supplementary Table3-0324.docx]

**Supplementary Table 3. The overlapped miRNAs of HSPA1A/HSPA2/HSPA8 predicted by TargetScan and miRDB**

| **Gene Symbol** | **Gene Description** | **miRNA Name** | **Position in the UTR** | **seed match** | **Target Score of miRDB** |
| --- | --- | --- | --- | --- | --- |
| HSPA1A | heat shock protein family A (Hsp70) member 1A | hsa-miR-6768-5p | 84-90 | 7mer-m8 | 84 |
| HSPA1A | heat shock protein family A (Hsp70) member 1A | hsa-miR-6754-3p | 111-117 | 7mer-m8 | 92 |
| HSPA1A | heat shock protein family A (Hsp70) member 1A | hsa-miR-412-3p | 111-117 | 7mer-m8 | 92 |
| HSPA1A | heat shock protein family A (Hsp70) member 1A | hsa-miR-223-5p | 196-202 | 7mer-m8 | 91 |
| HSPA1A | heat shock protein family A (Hsp70) member 1A | hsa-miR-4699-3p | 238-245 | 8mer | 91 |
| HSPA1A | heat shock protein family A (Hsp70) member 1A | hsa-miR-561-3p | 245-252 | 8mer | 90 |
| HSPA2 | heat shock protein family A (Hsp70) member 2 | hsa-miR-96-5p | 80-86,813-819 | 7mer-m8,7mer-1A | 89 |
| HSPA2 | heat shock protein family A (Hsp70) member 2 | hsa-miR-1271-5p | 80-86,813-819 | 7mer-m8,7mer-1A | 80 |
| HSPA2 | heat shock protein family A (Hsp70) member 2 | hsa-miR-4311 | 44-50,202-209,1314-1321,2271-2277 | 7mer-m8,8mer,8mer,7mer-m8 | 86 |
| HSPA2 | heat shock protein family A (Hsp70) member 2 | hsa-miR-3613-3p | 51-57,240-246,2115-2121,2509-2516 | 7mer-m8,7mer-1A,7mer-1A,8mer | 85 |
| HSPA2 | heat shock protein family A (Hsp70) member 2 | hsa-miR-5688 | 57-63,172-178,1135-1141 | 7mer-m8,7mer-m8,7mer-1A | 90 |
| HSPA2 | heat shock protein family A (Hsp70) member 2 | hsa-miR-495-3p | 57-63,172-178,1135-1141 | 7mer-m8,7mer-m8,7mer-1A | 90 |
| HSPA2 | heat shock protein family A (Hsp70) member 2 | hsa-miR-617 | 104-110 | 7mer-m8 | 82 |
| HSPA2 | heat shock protein family A (Hsp70) member 2 | hsa-miR-33a-3p | 185-192 | 8mer | 91 |
| HSPA2 | heat shock protein family A (Hsp70) member 2 | hsa-miR-3150a-5p | 195-201 | 7mer-m8 | 80 |
| HSPA2 | heat shock protein family A (Hsp70) member 2 | hsa-miR-3150b-5p | 195-201 | 7mer-m8 | 80 |
| HSPA2 | heat shock protein family A (Hsp70) member 2 | hsa-miR-586 | 209-215,424-431,1360-1367,2977-2983 | 7mer-m8,8mer,8mer,7mer-m8 | 97 |
| HSPA2 | heat shock protein family A (Hsp70) member 2 | hsa-miR-5700 | 210-216,2978-2985 | 7mer-m8,8mer | 82 |
| HSPA2 | heat shock protein family A (Hsp70) member 2 | hsa-miR-491-3p | 426-433,1856-1862 | 8mer,7mer-1A | 83 |
| HSPA8 | heat shock protein family A (Hsp70) member 8 | hsa-miR-26b-5p | 127-134 | 8mer | 91 |
| HSPA8 | heat shock protein family A (Hsp70) member 8 | hsa-miR-4465 | 127-134 | 8mer | 89 |
| HSPA8 | heat shock protein family A (Hsp70) member 8 | hsa-miR-1297 | 127-134 | 8mer | 92 |
| HSPA8 | heat shock protein family A (Hsp70) member 8 | hsa-miR-26a-5p | 127-134 | 8mer | 91 |
| HSPA8 | heat shock protein family A (Hsp70) member 8 | hsa-miR-301b-3p | 168-174 | 7mer-m8 | 85 |
| HSPA8 | heat shock protein family A (Hsp70) member 8 | hsa-miR-130a-3p | 168-174 | 7mer-m8 | 85 |
| HSPA8 | heat shock protein family A (Hsp70) member 8 | hsa-miR-4295 | 168-174 | 7mer-m8 | 86 |
| HSPA8 | heat shock protein family A (Hsp70) member 8 | hsa-miR-454-3p | 168-174 | 7mer-m8 | 85 |
| HSPA8 | heat shock protein family A (Hsp70) member 8 | hsa-miR-301a-3p | 168-174 | 7mer-m8 | 85 |
| HSPA8 | heat shock protein family A (Hsp70) member 8 | hsa-miR-130b-3p | 168-174 | 7mer-m8 | 85 |
| HSPA8 | heat shock protein family A (Hsp70) member 8 | hsa-miR-3666 | 168-174 | 7mer-m8 | 85 |
| HSPA8 | heat shock protein family A (Hsp70) member 8 | hsa-miR-20a-5p | 170-177 | 8mer | 98 |
| HSPA8 | heat shock protein family A (Hsp70) member 8 | hsa-miR-526b-3p | 170-177 | 8mer | 97 |
| HSPA8 | heat shock protein family A (Hsp70) member 8 | hsa-miR-93-5p | 170-177 | 8mer | 97 |
| HSPA8 | heat shock protein family A (Hsp70) member 8 | hsa-miR-519d-3p | 170-177 | 8mer | 97 |
| HSPA8 | heat shock protein family A (Hsp70) member 8 | hsa-miR-20b-5p | 170-177 | 8mer | 97 |
| HSPA8 | heat shock protein family A (Hsp70) member 8 | hsa-miR-106a-5p | 170-177 | 8mer | 97 |
| HSPA8 | heat shock protein family A (Hsp70) member 8 | hsa-miR-106b-5p | 170-177 | 8mer | 98 |
| HSPA8 | heat shock protein family A (Hsp70) member 8 | hsa-miR-17-5p | 170-177 | 8mer | 97 |
| HSPA8 | heat shock protein family A (Hsp70) member 8 | hsa-miR-221-3p | 16-23 | 8mer | 80 |
| HSPA8 | heat shock protein family A (Hsp70) member 8 | hsa-miR-222-3p | 16-23 | 8mer | 80 |
| HSPA8 | heat shock protein family A (Hsp70) member 8 | hsa-miR-3911 | 28-35 | 8mer | 87 |
| HSPA8 | heat shock protein family A (Hsp70) member 8 | hsa-miR-4307 | 39-45 | 7mer-m8 | 85 |
| HSPA8 | heat shock protein family A (Hsp70) member 8 | hsa-miR-205-5p | 47-53 | 7mer-1A | 81 |
| HSPA8 | heat shock protein family A (Hsp70) member 8 | hsa-miR-646 | 95-102 | 8mer | 86 |
| HSPA8 | heat shock protein family A (Hsp70) member 8 | hsa-miR-4524a-5p | 96-102 | 7mer-m8 | 81 |
| HSPA8 | heat shock protein family A (Hsp70) member 8 | hsa-miR-4524b-5p | 96-102 | 7mer-m8 | 81 |
| HSPA8 | heat shock protein family A (Hsp70) member 8 | hsa-miR-106a-3p | 166-172 | 7mer-m8 | 83 |
| HSPA8 | heat shock protein family A (Hsp70) member 8 | hsa-miR-519c-3p | 169-175 | 7mer-m8 | 94 |
| HSPA8 | heat shock protein family A (Hsp70) member 8 | hsa-miR-519a-3p | 169-175 | 7mer-m8 | 94 |
| HSPA8 | heat shock protein family A (Hsp70) member 8 | hsa-miR-519b-3p | 169-175 | 7mer-m8 | 94 |
| HSPA8 | heat shock protein family A (Hsp70) member 8 | hsa-miR-3609 | 171-177 | 7mer-1A | 82 |
| HSPA8 | heat shock protein family A (Hsp70) member 8 | hsa-miR-548ah-5p | 171-177 | 7mer-1A | 82 |
| HSPA8 | heat shock protein family A (Hsp70) member 8 | hsa-miR-4796-3p | 171-177 | 7mer-1A | 87 |
| HSPA8 | heat shock protein family A (Hsp70) member 8 | hsa-miR-4436b-5p | 193-200 | 8mer | 82 |
