# Supplementary material for: HSPA1A, HSPA2, and HSPA8 Are Potential Molecular Biomarkers for Prognosis among HSP70 Family in Alzheimer's Disease
Source: Dis Markers. 2022 Sep 30;2022:9480398. doi: 10.1155/2022/9480398 (PMC9553556; doi:10.1155/2022/9480398)
Supplement: Supplementary Materials — Supplementary Fig.1 Expression of three immune-related HSP70 family members in GSE132903. The blue box indicates the control group, and the orange box indicates the AD group. Data were analyzed by Student's T-test and expressed as the Mean ± SD. ∗P < 0.05; ∗∗∗P < 0.001. Supplementary Table 1. Immune molecules in the Immport database. Supplementary Table 2. Common TFs of the 3 hub genes from hTFtarget by jvenn. Supplementary Table 3. The overlapped miRNAs of HSPA1A/HSPA2/HSPA8 predicted by TargetScan and miRDB [file 9480398.f1.zip › Supporting Information-0824.docx]

**Supporting Information**





**Supplementary Fig.1** Expression of three immune-related HSP70 family members in GSE132903. The blue box indicates the control group, and the orange box indicates the AD group. Data were analyzed by Student’s T-test and expressed as the Mean ± SD. **P* < 0.05; ****P* < 0.001.
